# Comparison of the red blood cell indices based on accuracy, sensitivity, and specificity to predict one-year mortality in heart failure patients

**DOI:** 10.1186/s12872-022-02987-x

**Published:** 2022-12-07

**Authors:** Morteza Hosseinpour, Mohammad Reza Hatamnejad, Mohammad Nima Montazeri, Hamed Bazrafshan drissi, Ali Akbari Khezrabadi, Ehsan Shojaeefard, Shokoufeh Khanzadeh

**Affiliations:** 1 Faculty of Medicine, Shiraz University of Medical Sciences, Shiraz, Iran; 2 Department of Cardiology, Shiraz University of Medical Sciences, and Zand St, PO Box: 71348-14336, Shiraz, Iran; 3 Faculty of Medicine, I.M. Sechenov First Moscow State Medical University, Moscow, Russia; 4 Student Research Committee, Tabriz University of Medical Sciences, Tabriz, Iran

**Keywords:** Erythrocyte indices, Heart failure, Red cell distribution width, Hematocrit, Mean corpuscular hemoglobin, Mean corpuscular hemoglobin concentration, Mean corpuscular volume, Anemia

## Abstract

**Background:**

Various investigations have specified the role of each RBC indices separately [including hemoglobin (Hb), hematocrit (HCT), mean corpuscular volume (MCV), mean corpuscular hemoglobin (MCH), mean corpuscular hemoglobin concentration (MCHC), and red blood cell distribution width (RDW)] to predict the prognosis of acute heart failure (AHF) patients. However, in the current study, these variables were compared based on accuracy, sensitivity, and specificity to determine the best prognostic factor.

**Methods:**

Of 734 heart failure patients referred to the emergency department, 400 cases were enrolled based on the inclusion and exclusion criteria. Data of them were documented, and patients were followed for one year. Eventually, the association of clinical variables and RBC indices with one-year mortality was explored.

**Results:**

The study included 226 (56%) men and 174 (44%) women with a median age of 66 years. Body Mass Index (HR 1.098, *p* = 0.016), Hb (HR 0.728, *p* = 0.024), HTC (HR 0.875, *p* = 0.066), MCHC (HR 0.795, *p* = 0.037), and RDW-CV (HR 1.174, *p* = 0.006) were confirmed as predictors of long-term mortality. Despite confirming the predictive role of these variables by ROC curves, their sensitivity and specificity were reported as follows: [72% and 50% for Hb], [75% and 52% for  HCT], [88% and 27% for MCHC], and [49% and 81% for RDW]. In addition, stratified groups of patients, based on normal cut-off values obtained from scientific literature, had significantly different survival in Kaplan–Meier analyses.

**Conclusion:**

Whilst proving the predictive role of Hb,  HCT, MCHC, and RDW in AHF patients, the most sensitive measurement was MCHC and the most specific one was RDW; therefore, these variables should be considered for risk stratification purposes of AHF patients in daily clinical practice.

**Supplementary Information:**

The online version contains supplementary material available at 10.1186/s12872-022-02987-x.

## Introduction

Anemia and hematologic profile have been always taken into consideration to estimate the severity and prognosis of cardiac ailments such as heart failure [1, 2]. Several red blood cell (RBC) indices, including hemoglobin level (Hb) [3], hematocrit ( HCT) [4], mean corpuscular volume (MCV), mean corpuscular hemoglobin (MCH) [5], mean corpuscular hemoglobin concentration (MCHC) [6], and red blood cell distribution width (RDW) have been shown to predict the mortality or readmission to the hospital in acute heart failure (AHF) patients [7]. However, to the best of our knowledge, no one has compared them based on accuracy, sensitivity, and specificity to determine the best prognostic factor for heart failure patients. Thus, we conducted the study to determine which one of the RBC variables is more beneficial in daily clinical practice.

## Materials and methods

We conducted a prospective cohort study on the emergency department of Al-Zahra charity hospital, a university-affiliated tertiary medical center in Shiraz, Iran, from June 2019 to December 2020. All patients referred to the emergency department with symptoms of AHF were entered based on the following inclusion criteria and provided a form of consent to participate in the study.

This study included patients aged 18 years and above requiring (1) Hospitalization with a diagnosis of AHF according to the guidelines of the European Society of Cardiology as a rapid or gradual onset of signs and symptoms of heart failure, resulting in unplanned hospitalization and including new-onset AHF, without previously known cardiac dysfunction, and acute decompensation of chronic heart failure by two physicians, and (2) New York Heart Association (NYHA) classification of III or IV. Frequent admission of patients if they were referred more than once during the inclusion period was ignored and only the first admission was included in the database. Patients were excluded based on a history of severe aortic or mitral valvular disease, heart transplantation, active hematologic, oncologic, inflammatory disorders, severe renal dysfunction (GFR < 30 mL/min), and use of hemodialysis, blood transfusions, iron supplements, B12, and folic acid in the last 3 months.

Of 734 patients who were eligible for the study, 400 cases were qualified and enrolled in the study. Clinical assessment including age, sex, body mass index (BMI), medical history, social history, co-morbidities, NYHA classification, and medication history was applied at the baseline. The blood sample was sent for laboratory tests of complete blood count, hemostasis tests, cardiac biomarkers, lipid profile, electrolytes, and renal and hepatic function. Echocardiography was performed on each participant, according to the American Society of Echocardiography to determine ejection fraction (EF) values. Normal adult values of RBC measurements were obtained from scientific literature [RBC count: 4–4.5 (10^6/ µL), Hb: 12–16 (g/dL) for women and 13–18 (g/dL) for men, HCT 38–47 (%), MCV 82–92 (fL), MCH 28–32 (Pg), MCHC 32–36 (g/dL), and RDW: 12–16 (fL)] [8, 9]; consequently, they were considered as the bases for further classification (upper than normal range, normal range, and lower than normal range) and Kaplan-Meier analysis.

A phone call was made every 3 months since the patient was included, to evaluate endpoint occurrence. The endpoint was all-cause mortality during a one-year follow-up.

The study was performed in compliance with the international guidelines on clinical investigation of the World Medical Association’s Declaration of Helsinki; the university ethics committee approved the study protocol. Before the study, all patients gave written informed consent.

The statistical analysis was performed using the SPSS V.26.0 software package. Median and quartiles were used to describe continuous variables; however, categorical variables were represented by frequencies and percentages. Variables were compared concerning the occurrence of death (either in-hospital or long-term), using the Chi-square test, Student t-test, and the Mann–Whitney U test for categorical variables, continuous variables with normal distribution, and abnormal distributions, respectively. The association between all-cause mortality as the dependent variable and RBC indices as the independent variables was analyzed using univariate Cox proportional hazards regression analysis and quantified by hazard ratios, confidence interval, and statistical significance. Variables were included in the multivariable Cox proportional hazards model due to their significance in the univariate analysis or because they were considered clinically significant. Receiver operating characteristic (ROC) curves were applied to determine the accuracy, sensitivity, and specificity of RBC variables to predict one-year mortality. Patients were stratified based on normal adult values, and comparing the groups' survival was carried out by Kaplan–Meier curve and log-rank test. The results of all analyses were considered as significant if a P value of less than 0.05 was obtained.

## Results

**Fig. 1:**
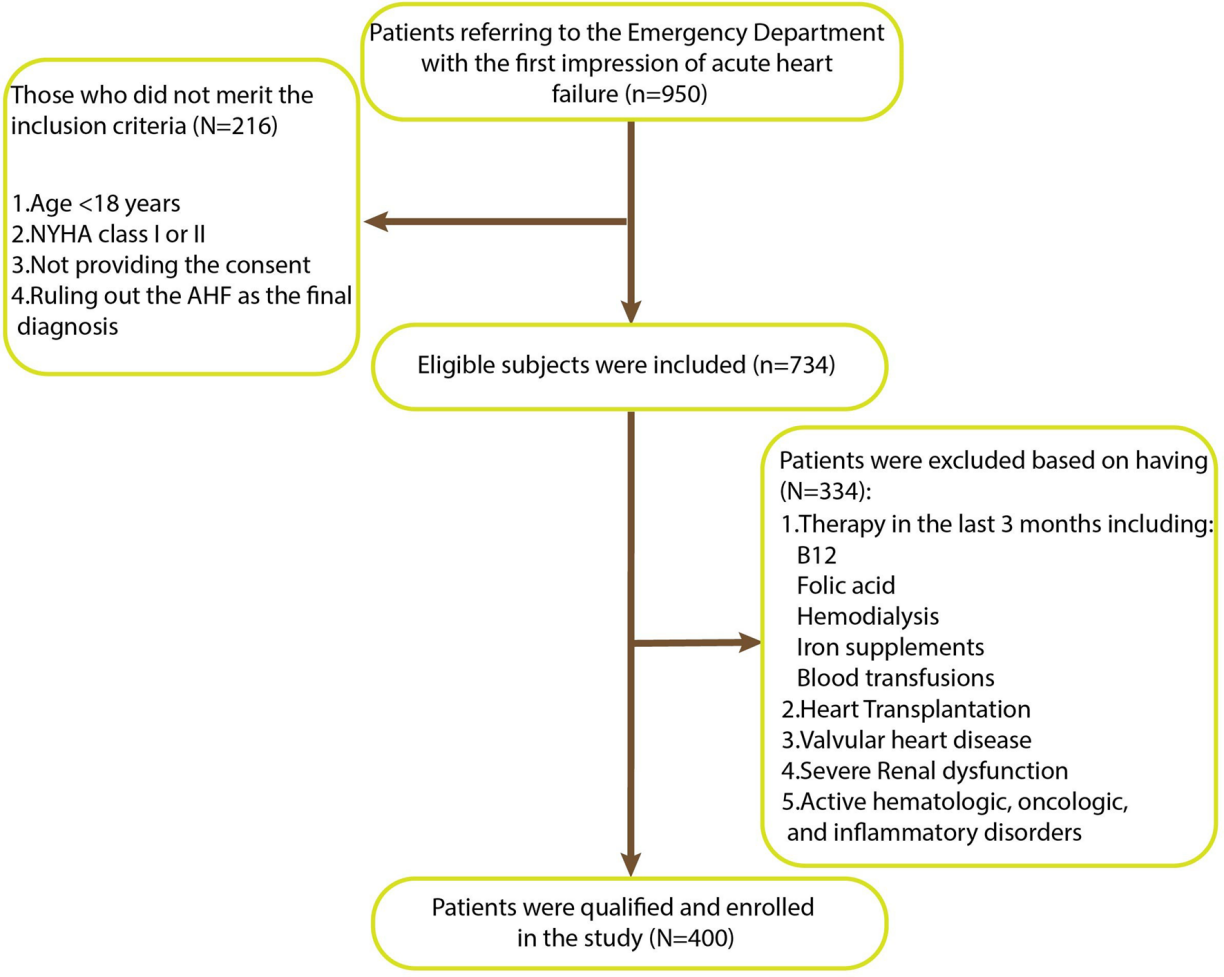
Flow chart of the patient selection

A total of 400 subjects were enrolled based on the inclusion and exclusion criteria (Fig. 1). The descriptive analysis of patients is shown in Table 1. The study included 226 (56%) men and 174 (44%) women participants with a median age of 66 years. In 123 (31%) patients, the current smoking was noted; 96 (24%) patients announced substance consumption, mainly opium, and this material can impact the heart or coronary vessels [10]. Median EF (30%) showed severe systolic dysfunction. The majority of patients fell within the normal range according to BMI (median:24, first and third quartiles: 22–27) and had NYHA classification III (54.5%). Dyspnea and diminished exercise capacity were the most common symptoms (98% and 84%). Rales/wheeze in 278 (69%) patients and diminished breath sound in 180 (45%) patients were the main signs in physical examination. Hypertension (61%), previous coronary artery disease (58%), and hyperlipidemia (51%) were the significant comorbidities. Ischemic heart disease was the most prominent cause of heart failure (32%). Patients mainly suffer from decompensated rather than de novo heart failure (68% vs. 32%). Most of the patients declared taking antiplatelet (71%), ß-Blocking agent (56%), and loop-diuretics (46%). Among 400 patients, 20 (5%) died during the hospital course and 380 (95%) patients were followed till the end of the study. In the meantime, 57(14%) patients expired with a median time to death of 3 months (Table 3). Comparison between the survivors and deceased (either in the hospital or long-term) was done using the Chi-square test, Student t-test, and the Mann-Whitney U test (Tables 1 and 2). The deceased were more likely than the survivors to have the anasarca (*p* = 0.01), a previous history of cerebrovascular disease (*p* = 0.02), diabetes mellitus (*p* = 0.01), and revascularization (*p* = 0.02). There was a remarkable difference in EF (*p* = 0.029), RBC count (*p* = 0.004), Hb (*p* < 0.001),  HCT (*p* < 0.001), MCH (*p* = 0.003), MCHC (*p* = 0.018), RDW-CV (*p* < 0.001), and uric acid (*p* < 0.001) between the two groups. Additionally, the comparison of variables between survivors and deceased groups of de novo and decompensated heart failure patients are illustrated in Additional file 1: Table S1 and Additional file 2: Table S2.

**Table 1:** Baseline characteristics of the patients based on the outcome

Characteristic	All patients ^a^ (n = 400)	Survivors ^b^ (n = 323, 81%)	Deceased ^b^ (n = 77, 19%)	*p*-value ^c^
Age, years	66 (57–76)	66 (57–75)	67 (56–78)	0.52
*Sex*				
Men	226 (56%)	186 (58%)	40 (52%)	0.37
Women	174 (44%)	137 (42%)	37 (48%)	
Current smoker	123 (31%)	94 (29%)	29 (38%)	0.14
Substance user	96 (24%)	77 (24%)	19 (25%)	0.87
BMI (kg/m^2^)	24 (22–27)	24 (22–27)	24(21–26)	0.11
*NYHA classification*				
NYHA III	218 (54.5%)	181 (56%)	37 (48%)	0.20
NYHA IV	182 (45.5%)	142 (44%)	40 (52%)	
Ejection fraction (%)	30 (20–40)	30 (20–40)	25 (15–35)	**0.02**
*Symptoms*				
Dyspnea	392 (98%)	317 (98%)	75 (97%)	0.67
Cough	79 (20%)	65 (20%)	14 (18%)	0.70
Diminished exercise capacity	335 (84%)	266 (82%)	69 (89%)	0.12
Orthopnea	189 (47%)	148 (46%)	41 (53.2%)	0.24
PND	45 (11%)	32 (10%)	13 (17%)	0.08
Peripheral edema	186 (46%)	145 (45%)	41 (53%)	0.18
*Signs*				
Elevated JVP	40 (10%)	29 (9%)	11 (14%)	0.16
Diminished breath sound	180 (45%)	142 (44%)	38 (49%)	0.39
Rales/Wheeze	278 (69%)	220 (68%)	58 (75%)	0.21
S_3_ or S_4_ heart sound	161 (40%)	129 (40%)	32 (41%)	0.13
TR or MR murmur	158 (39%)	124 (39%)	34 (44%)	0.35
Hepatomegaly	8 (2%)	5 (1.5%)	3 (3.9%)	0.18
Ascites	48 (12%)	34 (10%)	14 (18%)	0.06
Anasarca	33 (8%)	18 (5%)	15 (19%)	**0.01**
*Vital status*				
SBP (mm Hg)	130 (110–150)	130 (115–150)	120 (100–140)	0.30
DBP (mm Hg)	80 (70–90)	80 (70–90)	75 (64–85)	0.21
HR (per minute)	85 (75–98)	85 (75–95)	90 (77–107)	0.43
RR (per minute)	17 (16–18)	16 (15–18)	17 (16–19)	
O_2_ saturation (%)	95 (92–96)	95 (92–97)	94 (90–96)	0.83
*Past medical history*				
Diabetes mellitus	165 (41%)	124 (38%)	41 (53%)	**0.01**
Controlled *	55 (33%)	40 (32%)	15 (37%)	0.61
Uncontrolled *	110 (67%)	84 (68%)	26 (63%)	
Hypertension	245 (61%)	199 (62%)	46 (60%)	0.76
Controlled **	114 (46.5%)	90 (45%)	24 (52%)	0.39
Uncontrolled **	131(53.5%)	109 (55%)	22 (48%)	
Hyperlipidemia	203 (51%)	162 (50%)	41 (53%)	0.62
Hyperthyroidism	6 (1.5%)	3 (1%)	3 (4%)	0.08
Hypothyroidism	20 (5%)	16 (5%)	4 (5%)	0.93
Cerebrovascular disease	25 (6%)	16 (5%)	9 (12%)	**0.02**
Liver disease	13 (3%)	8 (3%)	5 (7%)	0.07
Asthma/COPD	53 (13%)	40 (12%)	13 (17%)	0.29
Previous coronary artery disease	231 (58%)	184 (57%)	47 (61%)	0.51
Revascularization (PCI or CABG)	156 (39%)	118 (36%)	38 (49%)	**0.02**
*Etiology of HF*				
Infection	76 (19%)	63 (20%)	13 (17%)	0.27
Hypertensive	69 (17%)	59 (18%)	10 (13%)	0.73
Cardiac arrhythmia	47 (12%)	37 (12%)	10 (13%)	0.76
Valvular heart disease	35 (9%)	25 (8%)	10 (13%)	0.14
Ischemic heart disease	128 (32%)	98 (30%)	30 (39%)	0.14
Dilated cardiomyopathy	45 (11%)	41 (12%)	4 (5%)	0.06
*Types of acute HF*				
De novo HF	128 (32%)	104 (32%)	24 (31%)	0.86
Decompensated HF	272 (68%)	219 (68%)	53 (69%)	
*Past Medication history*				
Antiplatelet	284 (71%)	224 (70%)	60 (78%)	0.13
Anticoagulation	111 (28%)	83 (26%)	28 (36%)	0.06
ACE inhibitor	81(20%)	69 (21%)	12 (16%)	0.25
ARB	121 (30%)	99 (31%)	22 (29%)	0.72
Calcium channel blocker	47 (12%)	40 (12%)	7 (9%)	0.42
ß-Blocking agent	224 (56%)	179 (55%)	45 (58%)	0.63
Loop diuretics	185 (46%)	149 (46%)	36 (47%)	0.92
Thiazide diuretics	11 (3%)	8 (2%)	3 (4%)	0.49
Potassium sparing diuretics	126 (31%)	99 (31%)	27 (35%)	0.11
Statins	196 (49%)	161 (50%)	35 (45%)	0.48
Fibrates	4 (1%)	4 (1%)	0 (0%)	0.42
Oral antidiabetic drugs	85 (21%)	69 (21%)	16 (21%)	0.91
Insulin	41 (10%)	29 (9%)	12 (16%)	0.08
Digitalis	67 (17%)	51 (16%)	16 (21%)	0.29
Nitrates	154 (38%)	123 (38%)	31 (40%)	0.72
Allopurinol	10 (2.5%)	6 (2%)	4 (5%)	0.09

*BMI* Body mass index; *NYHA* New York Heart Association Classification; *PND* Paroxysmal nocturnal dyspnea; *JVP* Jugular venous pulse; *TR* Tricuspid regurgitation; *MR* mitral regurgitation; *SBP* Systolic blood pressure; *DBP* Diastolic blood pressure; *HR* heart rate; *RR* respiratory rate; *COPD* Chronic obstructive pulmonary disease; *PCI* Percutaneous coronary intervention; *CABG* Coronary artery bypass grafting; *ADHF* Acute decompensated heart failure; *HF* Heart failure; *ACE* Angiotensin-converting enzyme; *ARB* Angiotensin receptor blocker

^a^ Binary variables were expressed by number (percentage); continuous variables were illustrated as Median (first quartile-third quartile)

^b^ Variables were compared using the Chi-square test, Student t test, and the Mann-Whitney U test for categorical variables, continuous variables with normal distribution, and non-normal distributions, respectively

^c^ All statistically significant p values (*p* < 0.05) are in bold

* They were measured in patients with diabetes mellitus

** They were measured in patients with hypertension

**Table 2:** Laboratory analysis

Laboratory Parameters	All patients ^a^ (n = 400)	Survivors ^b^ (n = 323, 81%)	Deceased ^b^ (n = 77, 19%)	* p-value* ^c^
Troponin I (Mic gr/L)	0.1 (0.1–0.1)	0.1 (0.1–0.1)	0.1 (0.1–0.1)	0.092
WBC count (10^3/µL)	7.4 (6–9)	7.3 (6–9)	8 (6–10)	0.142
RBC count (10^6/ µL)	4.4 (3.8–4.8)	4.4 (4–4.8)	3.9 (3.5–4.8)	**0.004**
Hemoglobin (g/dL)	12.0 (10.7–13.5)	12.5 (10.9–13.7)	11.2 (9.5–12.4)	**p < 0.001**
Hematocrit (%)	36 (32–40)	37 (33–40)	33 (30–37)	**P < 0.001**
MCV (f lit)	84 (78–89)	85 (78–89)	83 (78–87)	0.098
MCH (Pg)	28 (25–29)	28 (25–30)	27 (25–28)	**0.003**
MCHC (g/dL)	33 (32–34)	33 (32–34)	32 (31–33)	**0.018**
RDW-CV (fL)	14 (13–16)	14 (13–16)	16 (14–17)	**P < 0.001**
Platelet count (10^3/µL)	187 (155–231)	186 (155–231)	200 (152–235)	0.425
PT(sec)	13.8 (13–16)	13.7 (13–15)	14.8 (13–17)	0.161
PTT (sec)	33 (30–38)	33 (30–38)	34 (30–39)	0.244
INR (Index)	1.2 (1–1.5)	1.2 (1–1.4)	1.3 (1.1–1.7)	0.183
Random BS (mg/dL)	120 (96–173)	120 (95–170)	118 (101–183)	0.305
Sodium (mEq/dL)	139 (136–141)	139 (137–141)	137 (133–140)	0.347
Potassium (mEq/dL)	4.3 (4–4.6)	4.2 (4–4.6)	4.4 (4–5)	0.465
BUN (mg/dL)	21 (16–28)	20 (16–27)	21 (16–35)	0.121
Creatinine (mg/dL)	1.1 (0.9–1.4)	1.1 (0.9–1.3)	1.2 (1–1.4)	0.051
SGOT (mg/dL)*	22 (17–35) [150]	22 (17–32)	31 (18–57)	0.081
SGPT (IU/L)*	22 (14–38) [150]	20 (14–35)	29 (17–84)	0.064
ALP (mg/dL)*	188 (153–251) [144]	182 (150–248)	194 (176–270)	0.074
Albumin (mg/dL)*	3.9 (3.6–4.2) [134]	4 (3.7–4.2)	3.6 (3.3–4)	0.128
Globulin (g/dL)*	2.4 (2.1–2.9) [108]	2.4 (2.1–2.8)	2.9 (2.3–3.2)	0.506
Total protein (g/dL)*	6.5 (6-6.8) [108]	6.5 (6.1–6.8)	6.5 (5.8–6.8)	0.709
Total bilirubin (mg/dL)*	0.9 (0.5–1.3) [115]	0.8 (0.5–1.3)	1.15 (0.7–2.2)	0.075
Direct bilirubin (mg/dL)*	0.3 (0.2–0.5) [119]	0.3 (0.2–0.4)	0.5 (0.3–0.7)	0.064
Triglyceride (mg/dL)*	98 (71–121) [163]	100 (73–124)	84 (66–100)	0.053
Cholesterol (mg/dL)*	135 (104–164) [162]	138 (116–161)	109 (90–167)	0.145
HDL-CH (mg/dL)*	37 (30–43) [156]	38 (32–43)	36 (23–45)	0.293
LDL-C (mg/dL)*	74 (49–98) [156]	76 (50–98)	54 (48–95)	0.204
Uric acid (mg/dL)*	7.8 (5.6–9.5) [109]	7.0 (5.3–8.7)	9.8 (8.3–12.2)	**P < 0.001**
Ck-mb (IU/L)*	14 (11–20) [130]	13 (10–17)	17 (12–29)	0.152

*WBC* White blood cell ; *RBC* Red blood cells; *MCV* Mean corpuscular volume; *MCH* Mean corpuscular hemoglobin; *MCHC* Mean corpuscular hemoglobin concentration; *RDW* Red blood cell distribution width; *PT* Prothrombin time; *PTT* Partial thrombin time; *INR* international normalized ratio; *BS* Blood sugar; *BUN* Blood urea nitrogen; *SGOT* Serum glutamic oxaloacetic transaminase; *SGPT* Serum glutamic pyruvic transaminase; *ALP* Alkaline phosphatase; *HDL* High-density lipoprotein; *LDL* Low-density lipoprotein; *CK-MB* Creatine kinase-MB

^a^ Laboratory parameters were represented by Median (first quartile-third quartile)

^b^ Comparing the groups of patients was tested using the Mann–Whitney U test or the Student’s t-test depending on the distribution normality of the variables, and illustrated by Median (first quartile-third quartile)

^c^ All statistically significant* p* values (*p* < 0.05) are in bold

* Shows laboratory parameters that were not requested for all patients. The number of patients from which statistics was calculated is shown in a bracket in front of the quartiles

**Table 3:** The patients’ outcome

Outcome	Patients results
Days of admission	4 (2–5)
Survivors	323 (81%)
In-hospital mortality	20 (5%)
One-year mortality	57 (14%)
Times of follow up (months)	12 (12–12)
Time to death (months)	3 (1–5)

The outcome of the patients was presented as number (%) or median (first quartile-third quartile).

BMI (HR 1.092, 95% CI 1.017–1.219, *p* = 0.015), Hb (HR 0.878, 95% CI 0.782–0.986, *p* = 0.028),  HCT (HR 0.945, 95% CI 0.905–0.986, *p* = 0.010), MCH (HR 0.922, 95% CI 0.855–0.994, *p* = 0.033), MCHC (HR 0.761, 95% CI 0.640–0.905, *p* = 0.002), and RDW-CV (HR 1.202, 95% CI 1.101–1.312, *p* < 0.001) were proved as predictors of long-term mortality in univariate analysis of cox proportional hazards regression. Among them, only  HCT and MCH lost their statistical significance when they were analyzed by the multi-variate method of the Cox proportional hazard model (BMI [HR 1.098, 95% CI 1.018–1.186, *p* = 0.016], Hb [HR 0.728, 95% CI 0.553–0.958, *p* = 0.024], MCHC [HR 0.795, 95% CI 0.641–0.987, *p* = 0.037], RDW-CV [HR 1.174, 95% CI 1.046–1.317, *p* = 0.006], Table 4).

**Table 4:** Cox proportional hazard regression of time to long term mortality

Variables	Long-term mortality			
	Univariate analysis		Multivariate analysis	
	HR (95% CI) ^2^	*P* ^*1*^	HR (95% CI) ^2^	*P* ^*1*^
Age	1.017 (0.997; 1.036)	0.090	1.013 (0.993; 1.034)	0.206
Men*	0.970 (0.573; 1.640)	0.908	1.138 (0.644; 2.011)	0.655
BMI	1.092 (1.017; 1.219)	**0.015**	1.098 (1.018; 1.186)	**0.016**
Ejection fraction	0.985 (0.966; 1.005)	0.145	0.988 (0.966; 1.011)	0.297
SBP	0.993 (0.983; 1.003)	0.176	0.996 (0.984; 1.007)	0.476
Etiology of HF*		0.297		0.438
Infection	1.976 (0.535; 7.299)	0.307	1.565 (0.413; 5.935)	0.510
Hypertensive	2.115 (0.529; 8.456)	0.290	1.728 (0.421; 7.088)	0.448
Cardiac arrhythmia	3.275 (0.989; 10.84)	0.052	2.562 (0.752; 8.722)	0.132
Valvular heart disease	3.109 (0.778; 12.43)	0.109	2.805 (6.679; 11.58)	0.154
Ischemic heart disease	1.819 (0.483; 6.856)	0.377	1.442 (0.366; 5.678)	0.601
Type HF(De novo)*	0.898 (0.509; 1.584)	0.710	1.056 (0.580; 1.925)	0.858
RBC count	0.782 (0.552; 1.108)	0.166	0.841 (0.335; 1.347)	0.712
Hemoglobin	0.878 (0.782; 0.986)	**0.028**	0.728 (0.553; 0.958)	**0.024**
Hematocrit	0.945 (0.905; 0.986)	**0.010**	0.875 (0.759; 1.009)	0.066
MCV	0.982 (0.953; 1.012)	0.230	1.007 (0.952; 1.066)	0.805
MCH	0.922 (0.855; 0.994)	**0.033**	0.998 (0.798; 1.119)	0.916
MCHC	0.761 (0.640; 0.905)	**0.002**	0.795 (0.641; 0.987)	**0.037**
RDW-CV	1.202 (1.101; 1.312)	**p < 0.001**	1.174 (1.046; 1.317)	**0.006**

*BMI* Body mass index; *SBP* Systolic blood pressure; *HF* Heart failure; *RBC* Red blood cell; *MCV* Mean corpuscular volume; *MCH* Mean corpuscular hemoglobin; *MCHC* Mean corpuscular hemoglobin concentration; *RDW* Red blood cell distribution width.

All statistically significant p values (p < 0.05) are in bold.

^1^ Statistical significance of hazard ratio.

^2^ Hazard ratio calculated by multivariate Cox proportional hazard regression for long-term mortality and its 95% confidence interval.

* Women, dilated cardiomyopathy, and decompensated heart failure were considered the constant values for the sex, etiology, and type of heart failure, respectively.

**Fig. 2:**
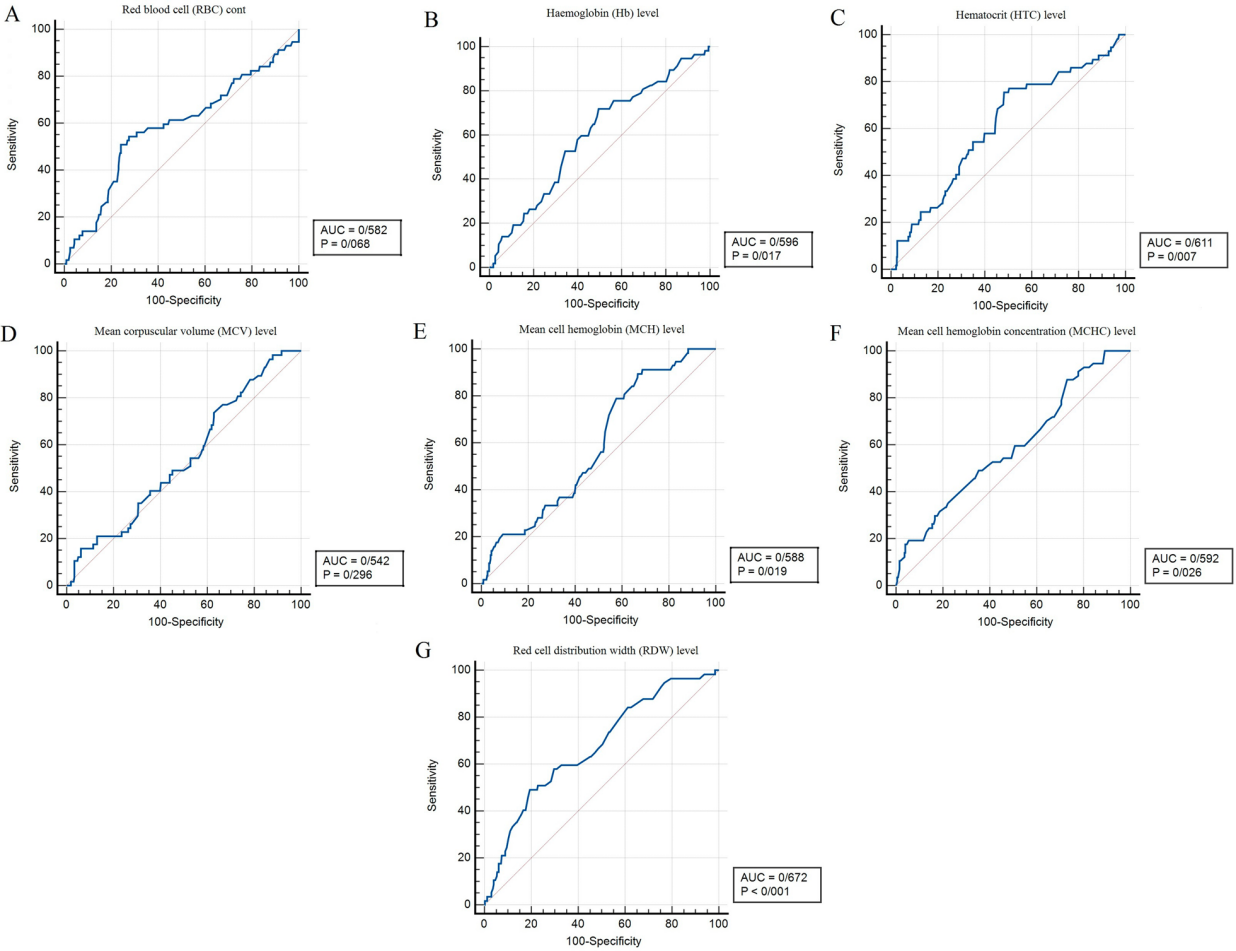
The receiver operating characteristic (ROC) curves analyses were applied to predict mortality within the one-year follow-up for **A** Red blood cell count, **B** Hemoglobin, **C** Hematocrit, **D** Mean corpuscular volume, **E** Mean corpuscular hemoglobin, **F** Mean corpuscular hemoglobin concentration, and **G** Red blood cell distribution width level

ROC curves for RBC indices and clinical variables to predict one-year mortality are illustrated in Fig. 2 and Additional file 3: Fig. S1, respectively; their analyses are provided in Table 5. Cut-off values [Area under the ROC Curve(AUC), 95%CI, *p*-value] were 72 years [0.569 (0.519–0.619), *p* = 0.102] for age, 22.3 kg/m^2^ [0.581 (0.531–0.630), *p* = 0.049] for BMI, 15% [0.562 (0.512–0.612), *p* = 0.157] for EF, 117 mmHg [0.561 (0.511–0.610), *p* = 0.171] for systolic blood pressure, 4.09 million cells/µL [0.582 (0.530–0.632), *p* = 0.068] for RBC count, 12.4 g/dL [0.596 (0.545–0.646), p = 0.017] for Hb, 37.1% [0.611 (0.560–0.660), *p* = 0.006] for  HCT, 87.7 fL [0.542 (0.490–0.593), *p* = 0.296] for MCV, 29.3 Pg [0.588 (0.537–0.638), *p* = 0.019] for MCH, 33.4 g/dL [0.592 (0.541–0.642), *p* = 0.026] for MCHC, and 16.4 fL [0.672 (0.622–0.719), p < 0.001] for RDW-CV. Sensitivity and specificity of these measurements to predict one-year mortality were reported as [44% and 69% for age], [46% and 74% for BMI], [35% and 81% for EF], [44% and 71% for systolic blood pressure], [55% and 72% for RBC count], [72% and 50% for Hb], [75% and 52% for  HCT], [74% and 37% for MCV], [89% and 33% for MCH], [88% and 27% for MCHC], and [49% and 81% for RDW].

**Table 5:** Receiver operating characteristic (ROC) curve analysis for long-term risk prediction

Parameters	AUC (95% CI)		*P* value		Cut-off point	Sensitivity (%)	Specificity (%)
Age	0.569 (0.519–0.619)		0.102		72 (years)	44	69
BMI	0.581 (0.531–0.630)		**0.049**		22.3 (kg/m^2^)	46	74
Ejection fraction	0.562 (0.512–0.612)		0.157		15 (15%)	35	81
SBP	0.561 (0.511–0.610)		0.171		117 (mm Hg)	44	71
RBC count	0.582 (0.530–0.632)		0.068		4.09 (10^6/ µL)	55	72
Hemoglobin	0.596 (0.545–0.646)		**0.017**		12.4 (g/dL)	72	50
Hematocrit	0.611 (0.560–0.660)		**0.006**		37.1 (%)	75	52
MCV	0.542 (0.490–0.593)		0.296		87.7 (fL)	74	37
MCH	0.588 (0.537–0.638)		**0.019**		29.3 (Pg)	89	33
MCHC	0.592 (0.541–0.642)		**0.026**		33.4 (g/dL)	88	27
RDW-CV	0.672 (0.622–0.719)		**P < 0.001**		16.4 (fL)	49	81

*AUC* Area under the curve; *CI* Confidence interval; *BMI* Body mass index; *SBP* Systolic blood pressure; *RBC* Red blood cell; *MCV* Mean corpuscular volume; *MCH* Mean corpuscular hemoglobin; *MCHC* Mean corpuscular hemoglobin concentration; *RDW* Red bloodcell distribution width.

* All statistically significant p values (p < 0.05) are in bold

**Fig. 3:**
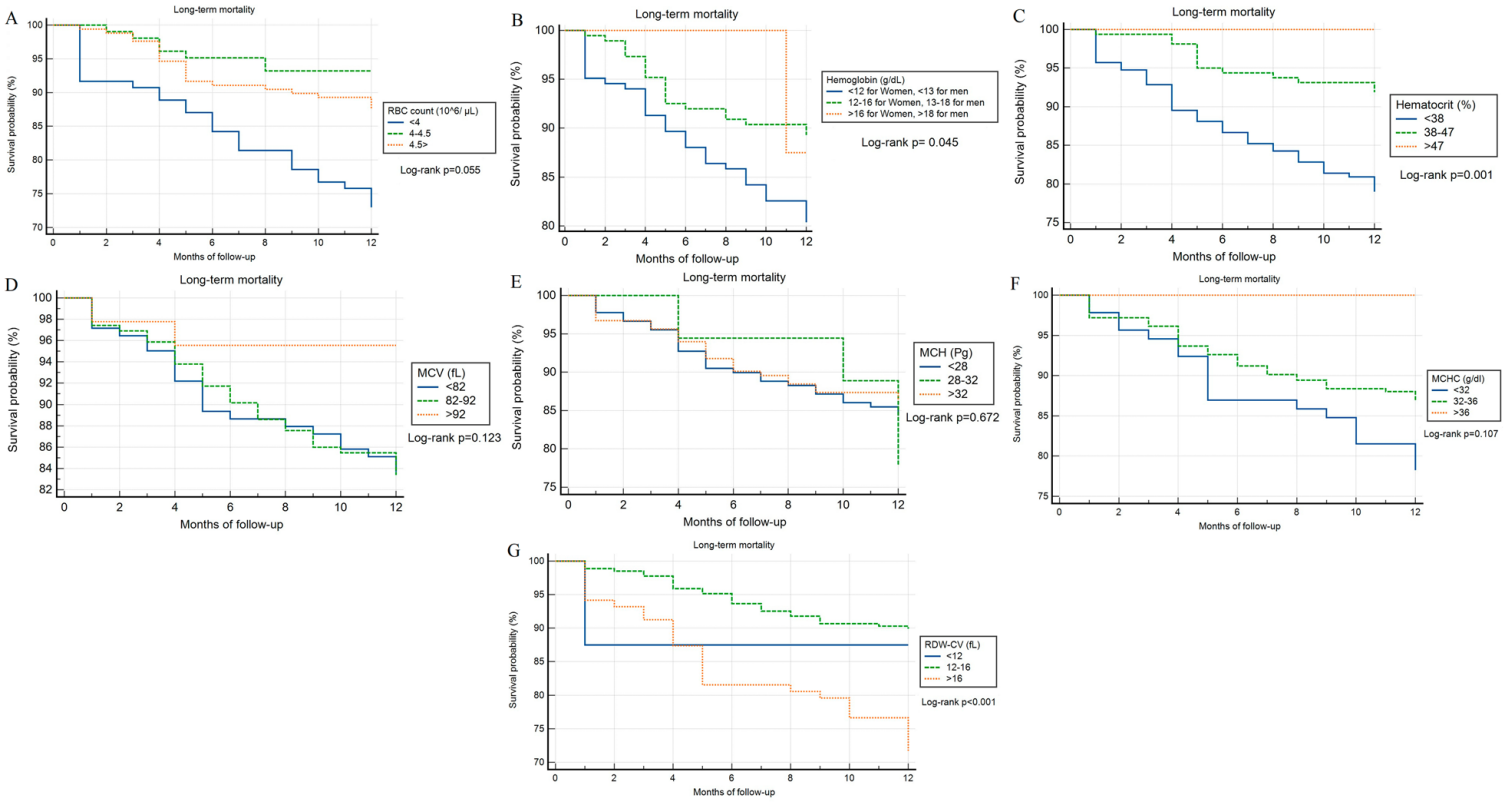
Kaplan–Meier curves and log-rank tests compared the tertile groups' survivals, which were categorized based on normal adult values, during the One-year follow-up for **A** Red blood cell count, **B** Hemoglobin, **C** Hematocrit, **D** Mean corpuscular volume, **E** Mean corpuscular hemoglobin, **F** Mean corpuscular hemoglobin concentration, and **G** Red blood cell distribution width.

RBC measurements were categorized based on the cut-off points (normal adult values) mentioned in the method section. The groups' survival was compared via Kaplan–Meier curves and log-rank. Log-rank of survival analysis for one-year follow-up within the tertile groups were described as *p* = 0.055 for RBC count, *p* = 0.045 for Hb, *p* = 0.001 for  HCT, *p* = 0.123 for MCV, *p* = 0.672 for MCH, *p* = 0.107 for MCHC, and *p* < 0.001 for RDW (Fig. 3).

## Discussion

Previous studies investigated the role of each RBC variable to determine the prognosis of heart failure patients separately [3, 4, 11–14], but a comparative study of RBC indices in terms of sensitivity, specificity, and accuracy to determine the best prognostic predictor has not been done yet. In the present study, whilst proving the predictive role of Hb,  HCT, MCHC, and RDW in heart failure patients, the most sensitive variable was MCHC and the most specific one was RDW.

### RBC count

Contrary to the other measurements, RBC count is the variable that is not influenced by the plasma alteration effect in the setting of heart failure [15]; thus it may not change significantly. Following previous studies, RBC count was not found as an independent predictor.

### Hemoglobin

There are different physiologic compensatory mechanisms for low Hb levels. One of them is increasing the cardiac output to maintain proper oxygen delivery to different organs, so this cardiac overloading will lead to heart failure in the future. Thus, it is known that a low level of Hb in heart failure patients can play a part in decompensation and a worse prognosis [16]. Okuno et al. [3] found that the Hb level at the time of AHF diagnosis in patients with preserved EF was an independent factor in predicting mortality for both men and women. On the other hand, Abebe et al. [11] divided severe heart failure patients into two anemic and non-anemic groups based on their Hb level, and Kaplan-Meier diagrams did not show a significant discrepancy in survival rate between the mentioned groups. Therefore, the role of Hb as a prognosis predictor in heart failure patients is contradictory. Predicting the role of Hb in the mortality of patients with AHF was illustrated by multivariate analysis. The current investigation showed appropriate sensitivity and slightly acceptable specificity with a remarkable size effect for predicting one-year mortality. The Kaplan-Meier analysis showed that the groups of patients which were divided based on Hb normal adult values had significantly different survival rates.

### Hematocrit

Blood oxygen content decreases by declining  HCT.  HCT is a determinant of blood viscosity. Hemodilution reduces the oxygen content and the viscosity of the blood, thereby increasing regional blood flow and cardiac output [17]. Hemodilution affects patients with heart failure as it results in impaired peripheral oxygen delivery. Compensatory mechanisms to evade tissue hypoxia include an increase in cardiac output by sympathetic stimulation, redistribution of blood flow, an increase in whole-body oxygen extraction ratio, and activation of aortic chemoreceptors with an increase in venomotor tone [18]. Achievement of hemoconcentration in hospitalized AHF patients showed to have better survival, compared to hemodilution [19]. Ling et al. [20] showed that plasma volume, which depends on weight and  HCT, was a predictor for prognosis in heart failure patients so  HCT was also associated with prognosis in heart failure patients. Guglin et al. [4] stated that a low level of Hb reduced  HCT and decreased blood concentration and viscosity, so stroke volume would increase, but this does not affect the prognosis of heart failure. The paper presented by Oczan Cetin et al. [21] reported a direct relationship between blood viscosity and the prognosis of patients with heart failure. In this study,  HCT has been illustrated to own a prognostic role in determining the mortality of patients with heart failure. The sensitivity of this indicator was acceptable, although its specificity was barely noticeable.

Plasma volume may increase in patients with decompensated heart failure, which exacerbates the prognosis; besides,  HCT and Hb, contrary to RBC count, are indirectly affected by plasma volume. Thus, this fact provides a base to justify different predicted results of these variables. Opposed to the RBC count,  HCT and Hb are adjusted to the plasma volume, indicating the prognosis predicting utility [15, 22, 23].

### MCV

Mean corpuscular volume is the measure of the average size of the circulatory erythrocyte, and it is principally used as an index for the differential diagnosis of anemia. Recently, MCV has been associated with mortality in many clinical settings [13]. Wolowiec et al. [5] found that there was no statistically significant relationship between MCV and the prognosis of patients with heart failure with 1-year follow-up. In our study, no mortality-predicting role can be assumed for MCV in heart failure patients.

### MCH

Mean corpuscular hemoglobin represents the average amount of Hb in RBCs, and Hb is essential for the distribution and delivery of oxygen to the tissues [24]. Following a study by Wolowiec et al. [5] that showed MCH was not a prognostic factor in heart failure patients, the current project failed to determine the MCH as a 1-year mortality predictor. Although it possesses an acceptable significant sensitivity in ROC analysis, in the multivariate model, with the effect of other co-factors, the prognostic utility was alleviated, and it cannot be supposed as a predictor.

### MCHC

MCHC is a measure of the concentration of hemoglobin per volume of packed RBCs. If the reduced hemoglobin synthesis rate is faster than the reduced synthetic RBC volume, then the MCHC level is decreased. Low MCHC, therefore, represents a gross estimate of the presence of relative hypochromia. MCHC provides information on the hemoglobin concentration of each RBC. If it decreases for a long period, the organs' oxygenation will reduce [6, 25]. Different mechanisms play a part in hypochromia. First of all, there is the probability of the existence of an issue with availability or adhesion of iron into Hb. Other mechanisms might be related to renal insufficiency, where the underlying renal disease causes erythropoietin insufficiency or resistance. Also, there is a possibility of a dilution effect, because changes in osmotic pressures in the setting of congestion may theoretically affect the relative concentration of hemoglobin within the erythrocyte [6]. Simbaqueba et al. [6] reported that hypochromia, which reflects the low level of MCHC, was associated with a worse prognosis in heart failure patients. Hammadah et al. [26] mentioned MCHC as an independent predictor of poor prognosis in patients with heart failure. On the other hand, in the study by Wolowiec [5], this determining role was rejected. In the current project, MCHC was identified as an independent factor in predicting the prognosis of patients with AHF. Despite the low level of specificity, the highest sensitivity makes this index more profitable among all RBC indices.

### RDW-CV

Several physiological and pathological conditions may impair erythropoiesis and, hence, promote a higher degree of heterogeneity of RBC volumes. This process is characterized by the variability in the size of circulating erythrocytes, which is conventionally known as anisocytosis. In patients with heart failure, the presence of anisocytosis may be interpreted as a homeostatic response to the disease, thus reflecting the existence of a potential link between ineffective erythropoiesis and chronic inflammation [27, 28]. Nutritional deficiencies are the other reason for anisocytosis as they are involved in the onset and progression of heart failure [29]. Progressive renal dysfunction is another major cause of anemia and anisocytosis, but it is also an important indicator of poor outcomes in heart failure patients. Anisocytosis also increases with aging as the result of numerous metabolic dysfunction. On the other hand, advanced age is also an effective factor for cardiac dysfunction [29]. Therefore, these facts show that heart failure and anisocytosis are common in many pathogenic processes. Nonetheless, anisocytosis can directly result in the onset and progression of heart failure. Anisocytosis leads to reduced oxygen delivery to the peripheral tissues; also, abnormal RBCs may play a part in the pathogenesis of cardiac fibrosis by amplifying inflammation, stress of cardiomyocytes, and apoptosis [29]. Different studies have proven the RDW role in prediction of heart failure prognosis [5, 7, 14, 30–32]. In current perusal, the highest effect size (hazard ratio), specificity, and accuracy for determining the long-term mortality risk in heart failure patients indicate RDW advantageous rather than other RBC indices.

### **Limitations**

This study reached its goal of evaluating the prognostic role of RBC markers in heart failure patients; however, there were also a few limitations. Conducting the project in one center might have influenced the external validity. Determining the type of anemia based on (Hb or MCV) and its relationship with groups of patients was not carried out. The iron profile of AHF patients was not included as an influential factor during this study. Also, other prognostic factors such as electrocardiogram changes in abnormalities were not evaluated.

## **Conclusion**

By determining the prognosis in patients with heart failure, it is possible to identify high-risk patients for initial interventions, which can reduce the rate of readmission, mortality, and medical system costs. In the present study, the role of RBC indices in determining the prognosis (one-year mortality) of heart failure patients was investigated. Among these, except for RBC, MCH, and MCV, all other measurements had a statistically significant relationship with the prognosis of patients. MCHC and RDW were the most sensitive and specific variables of RBC, respectively, used to obtain the prognosis of heart failure patients; they can be used in daily clinical workups to determine the risk of cardiovascular mortality in patients with AHF.

## Supplementary Information

**Additional file 1: Table S1.** Characteristics of the De novo heart failure patients

**Additional file 2: Table S2.** Characteristics of the Decompensated heart failure patients

**Additional file 3: Fig. S1.** The receiver operating characteristic (ROC) curves analyses were applied to predict mortality within the one-year follow-up for (A) Age, (B) Body mass index, (C) Ejection fraction, and (D) Systolic blood pressure

## Data Availability

Regarding the participants’ privacy the datasets used and/or analyzed during the current study are only available from the corresponding author on reasonable request.

## Abbreviations

RBC: Red blood cell
Hb: Hemoglobin
HCT: Hematocrit
MCV: Mean corpuscular volume
MCH: Mean corpuscular hemoglobin
MCHC: Mean corpuscular hemoglobin concentration
RDW: Red blood cell distribution width
AHF: Acute heart failure
NYHA: New York Heart Association
BMI: Body mass index
EF: Ejection fraction
ROC: Receiver operating characteristic
AUC: Area under the curve
