# Supplementary material for: Comparison of the red blood cell indices based on accuracy, sensitivity, and specificity to predict one-year mortality in heart failure patients
Source: BMC Cardiovasc Disord. 2022 Dec 7;22:532. doi: 10.1186/s12872-022-02987-x (PMC9727904; doi:10.1186/s12872-022-02987-x)
Supplement: Supplementary file 1 — Additional file 1: Table S1. Characteristics of the De novo heart failure patients [file 12872_2022_2987_MOESM1_ESM.docx]

| **Supplementary Table 1: Characteristics of the De novo heart failure patients** | | | | | |
| --- | --- | --- | --- | --- | --- |
| P-value^c^ | Deceased ^b^ | Survivors ^b^ | All patients ^a^ | Characteristic | |
|  | (n=24, 19%) | (n=104, 81%) | (n=128) |  | |
| 0.889 | 69 (55-77) | 67 (60-75) | 67 (59-75) | Age, years | |
| **0.007** | 6 (25%) | 58 (56%) | 64 (50%) | Men | Sex |
|  | 18 (75%) | 46 (44%) | 64 (50%) | Women |  |
| 0.353 | 10 (42%) | 33 (32%) | 43 (34%) | Current smoker | |
| 0.738 | 5 (21%) | 25 (24%) | 30 (23%) | Substance user | |
| 0.985 | 24 (20-27) | 24 (22-27) | 24 (22-27) | BMI (kg/m2) | |
| **0.016** | 12 (50%) | 78 (75%) | 90 (70%) | NYHA III | NYHA Class |
|  | 12 (50%) | 26 (25%) | 38 (30%) | NYHA IV |  |
| 0.294 | 30 (22-42) | 35 (20-50) | 35 (20-45) | Ejection fraction (%) | |
|  |  |  |  | **Past medical history** | |
| 0.076 | 14 (58%) | 40 (38%) | 54 (42%) | Diabetes mellitus | |
| 0.655 | 3 (21%) | 11 (27%) | 14 (26%) | Controlled * | |
|  | 11 (79%) | 29 (73%) | 40 (74%) | Uncontrolled * | |
| 0.610 | 17 (71%) | 68 (65%) | 85 (66%) | Hypertension | |
| 0.323 | 9 (53%) | 27 (40%) | 36 (42%) | Controlled ** | |
|  | 8 (47%) | 41 (60%) | 49 (58%) | Uncontrolled ** | |
| 0.462 | 14 (58%) | 52 (50%) | 66 (52%) | Hyperlipidemia | |
| **0.021** | 3 (12%) | 1 (1%) | 4 (3%) | Hyperthyroidism | |
| 0.846 | 2 (8%) | 10 (10%) | 12 (9%) | Hypothyroidism | |
| 0.235 | 2 (8%) | 3 (3%) | 5 (4%) | Cerebrovascular disease | |
| 0.569 | 1 (4%) | 3 (3%) | 4 (3%) | Liver disease | |
| 0.124 | 5 (21%) | 10 (10%) | 15 (12%) | Asthma/COPD | |
| 0.548 | 15 (62%) | 58 (56%) | 73 (57%) | Previous coronary artery disease | |
| 0.933 | 10 (42%) | 38 (36%) | 48 (37%) | Revascularization (PCI or CABG) | |
|  |  |  |  | **Etiology of HF** | |
| 0.807 | 3 (12%) | 15 (14%) | 18 (14%) | Infection | |
| 0.494 | 4 (17%) | 24 (23%) | 28 (22%) | Hypertensive | |
| 0.241 | 4 (17%) | 9 (9%) | 13 (10%) | Cardiac arrhythmia | |
| 0.782 | 2 (8%) | 7 (7%) | 9 (7%) | Valvular heart disease | |
| 0.790 | 9 (38%) | 36 (35%) | 45 (35%) | Ischemic heart disease | |
| 0.567 | 2 (8%) | 13 (12%) | 15 (12%) | Dilated cardiomyopathy | |
|  |  |  |  | **Past Medication history** | |
| 0.192 | 19 (79%) | 68 (65%) | 87 (68%) | Antiplatelet | |
| 0.129 | 7 (29%) | 15 (14%) | 22 (17%) | Anticoagulation | |
| 0.566 | 3 (12%) | 18 (17%) | 21 (16%) | ACE inhibitor | |
| 0.879 | 8 (33%) | 33 (32%) | 41 (32%) | ARB | |
| 0.122 | 1 (4%) | 17 (16%) | 18 (14%) | Calcium channel blocker | |
| 0.451 | 11 (46%) | 39 (37%) | 50 (39%) | ß-Blocking agent | |
| - | - | - | - | Loop diuretics | |
|  | - | - | - | Thiazide diuretics | |
|  | - | - | - | Potassium sparing diuretics | |
| **0.023** | 5 (21%) | 48 (46%) | 53 (41%) | Statins | |
| - | - | - | - | Fibrates | |
| 0.385 | 4 (17%) | 26 (25%) | 30 (23%) | Oral antidiabetic drugs | |
| **0.019** | 4 (16%) | 4 (4%) | 8 (6%) | Insulin | |
| 0.281 | 1 (4%) | 12 (11%) | 13 (10%) | Digitalis | |
| 0.790 | 9 (37%) | 36 (35%) | 45 (35%) | Nitrates | |
| 0.494 | 0 (0%) | 2 (2%) | 2 (1%) | Allopurinol | |
|  |  |  |  | **Laboratory parameters** | |
| 0.512 | 0.1 (0.1-0.115) | 0.1 (0.1-0.1) | 0.1 (0.1-0.1) | Troponin I (Mic gr/L) | |
| **P<0.001** | 9.3 (7.6-12.3) | 7.3 (5.5-9.3) | 7.5 (5.8-9.6) | WBC count (10^3/µL) | |
| 0.101 | 4 (3.6-4.7) | 4.4 (4-4.8) | 4.4 (3.9-4.8) | RBC count (10^6/ µL) | |
| **0.006** | 11.0 (9.9-12.5) | 13.0 (10.9-13.9) | 12.6 (10.9-13.7) | Hemoglobin (g/dL) | |
| 0.006 | 34 (30-37) | 38 (34-41) | 37 (33-41) | Hematocrit (%) | |
| 0.171 | 83 (80-85) | 85 (79-90) | 84 (80-89) | MCV (f lit) | |
| 0.163 | 27 (26-28) | 28 (26-30) | 28 (26-30) | MCH (Pg) | |
| 0.301 | 33 (32-34) | 33 (32-34) | 33 (32-34) | MCHC (g/dl) | |
| **0.006** | 15 (14-18) | 14 (13-15) | 14 (13-16) | RDW-CV (fl) | |
| 0.236 | 210 (162-248) | 188 (155-219) | 190 (156-222) | Platelet count (10^3/µL) | |
| 0.797 | 13.9 (13.4-15.5) | 13 (12.7-14.8) | 13.4 (12.8-15) | PT(sec) | |
| 0.867 | 33.5 (30-38) | 33 (30-37) | 33 (30-37) | PTT (sec) | |
| 0.855 | 1.2 (1.1-1.4) | 1.1 (1-1.3) | 1.12 (1.01-1.38) | INR (Index) | |
| **0.025** | 157 (105-305) | 119 (95-182) | 122 (96-184) | Random BS (mg/dL) | |
| 0.073 | 138 (134-140) | 139 (137-140) | 138 (137-140) | Sodium (mEq/dL) | |
| 0.798 | 4.2 (3.9-4.6) | 4.2 (4-4.5) | 4.2 (4-4.5) | Potassium (mEq/dL) | |
| 0.809 | 18 (15-24) | 19 (14-24) | 19 (14-24) | BUN (mg/dL) | |
| 0.557 | 1.1 (0.8-1.2) | 1 (0.9-1.2) | 1 (0.9-1.2) | Creatinine (mg/dL) | |
| 0.219 | 18 (14-102) | 20 (15-32) | 19 (15-33)[60] | SGOT (mg/dL) *** | |
| 0.352 | 15 (12-69) | 18.5 (15-27) | 18 (13-29)[60] | SGPT (IU/L) *** | |
| **0.033** | 218 (188-286) | 166 (142-200) | 182 (146-202)[58] | ALP (mg/dL) *** | |
| 0.115 | 3.7 (3.2-4.2) | 4.1 (3.7-4.4) | 4.1 (3.7-4.3)[58] | Albumin (mg/dL) *** | |
| 0.302 | 2.5 (2.3-3) | 2.3 (2.1-2.5) | 2.4 (2.1-2.6)[51] | Globulin (g/dL) *** | |
| 0.090 | 6.5 (5.9-6.8) | 6.5 (6.2-6.8) | 6.5 (6.2-6.8)[51] | Total protein (g/dL) *** | |
| 0.250 | 1.1 (0.7-1.3) | 0.8 (0.5-0.9) | 0.8 (0.5-1)[51] | Total bilirubin (mg/dL) *** | |
| 0.254 | 0.36 (0.32-0.66) | 0.26 (0.2-0.3) | 0.29 (0.2-0.3)[53] | Direct bilirubin (mg/dL) *** | |
| 0.462 | 99 (82-100) | 96 (69-120) | 98 (69-120)[62] | Triglyceride (mg/dL) *** | |
| 0.335 | 165.5 (133-180) | 134 (111-175) | 142 (116-177)[62] | Cholesterol (mg/dL) *** | |
| 0.627 | 39 (37-57) | 40 (35-47) | 40 (35-47)[62] | HDL-CH (mg/dL) *** | |
| 0.791 | 93 (71-104) | 84 (50-100) | 86 (59-104)[62] | LDL-C (mg/dL) *** | |
| 0.684 | 6.4 (5.4-8.9) | 6.4 (4.9-8.0) | 6.4 (5.2-8.3) [42] | Uric acid (mg/dL) *** | |
| 0.121 | 17 (13-28) | 12 (9-15) | 13 (10-19)[43] | Ck-mb (IU/L) ******* | |
| For abbreviations, please see Tables 1 and 2.  ^a^ Binary variables are expressed by number (percentage); continuous variables are illustrated as Median (first quartile-third quartile)  ^b^ Variables were compared using the Chi-square test, Student t test, and the Mann-Whitney U test for categorical variables, continuous variables with normal distribution, and non-normal distributions, respectively.  ^c^ All statistically significant p values (p < 0.05) are in bold.  * They were measured in patients with diabetes mellitus.  ** They were measured in patients with hypertension.  *** Shows laboratory parameters that were not requested for all patients. The number of patients from which statistics was calculated is shown in a bracket in front of the quartiles. | | | | | |
