# Supplementary material for: Comparison of the red blood cell indices based on accuracy, sensitivity, and specificity to predict one-year mortality in heart failure patients
Source: BMC Cardiovasc Disord. 2022 Dec 7;22:532. doi: 10.1186/s12872-022-02987-x (PMC9727904; doi:10.1186/s12872-022-02987-x)
Supplement: Supplementary file 2 — Additional file 2: Table S2. Characteristics of the Decompensated heart failure patients [file 12872_2022_2987_MOESM2_ESM.docx]

| **Supplementary Table 2: Characteristics of the Decompensated heart failure patients** | | | | | |
| --- | --- | --- | --- | --- | --- |
| P-value^c^ | Deceased ^b^ | Survivors ^b^ | All patients ^a^ | Characteristic | |
|  | (n=53, 19%) | (n=219, 81%) | (n=272) |  | |
| 0.582 | 65 (56-76) | 67 (57-78) | 65 (56-76) | Age, years | |
| 0.448 | 34 (64%) | 128 (58%) | 162 (60%) | Men | Sex |
|  | 19 (36%) | 91 (42%) | 110 (40%) | Women |  |
| 0.252 | 19 (36%) | 61 (28%) | 80 (29%) | Current smoker | |
| 0.684 | 14 (26%) | 52 (24%) | 66 (24%) | Substance user | |
| **0.048** | 24 (22-27) | 24 (21-26) | 24 (22-27) | BMI (kg/m2) | |
| 0.986 | 25 (47%) | 103 (47%) | 144 (53%) | NYHA III | NYHA Class |
|  | 28 (53%) | 116 (53%) | 128 (47%) | NYHA IV |  |
| **0.043** | 25 (20-35) | 20 (15-31) | 25 (20-35) | Ejection fraction (%) | |
|  |  |  |  | **Past medical history** | |
| 0.094 | 27 (51%) | 84 (38%) | 111 (41%) | Diabetes mellitus | |
| 0.353 | 12 (44%) | 29 (34%) | 41 (37%) | Controlled * | |
|  | 15 (56%) | 55 (66%) | 70 (63%) | Uncontrolled * | |
| 0.498 | 29 (55%) | 131 (60%) | 160 (59%) | Hypertension | |
| 0.723 | 15 (52%) | 63 (48%) | 78 (49%) | Controlled ** | |
|  | 14 (48%) | 68 (52%) | 82 (51%) | Uncontrolled ** | |
| 0.926 | 27 (51%) | 110 (50%) | 137 (50%) | Hyperlipidemia | |
| 0.485 | 0 (0%) | 2 (1%) | 2 (1%) | Hyperthyroidism | |
| 0.689 | 2 (4%) | 6 (3%) | 8 (3%) | Hypothyroidism | |
| 0.069 | 7 (13%) | 13 (6%) | 20 (7%) | Cerebrovascular disease | |
| 0.055 | 4 (7.5%) | 5 (2%) | 9 (3%) | Liver disease | |
| 0.793 | 8 (15%) | 30 (14%) | 38 (14%) | Asthma/COPD | |
| 0.707 | 32 (60%) | 126 (57%) | 158 (58%) | Previous coronary artery disease | |
| **0.009** | 28 (53%) | 80 (37%) | 108 (40%) | Revascularization (PCI or CABG) | |
|  |  |  |  | **Etiology of HF** | |
| 0.627 | 10 (19%) | 48 (22%) | 58 (21%) | Infection | |
| 0.395 | 6 (11%) | 35 (16%) | 41 (15%) | Hypertensive | |
| 0.708 | 6 (11%) | 28 (13%) | 34 (13%) | Cardiac arrhythmia | |
| 0.127 | 8 (15%) | 18 (8%) | 26 (10%) | Valvular heart disease | |
| 0.109 | 21 (40%) | 62 (28%) | 83 (30%) | Ischemic heart disease | |
| 0.060 | 2 (4%) | 28 (13%) | 30 (11%) | Dilated cardiomyopathy | |
|  |  |  |  | **Past Medication history** | |
| 0.371 | 41 (77%) | 156 (71%) | 197 (72%) | Antiplatelet | |
| 0.233 | 21 (40%) | 68 (31%) | 89 (33%) | Anticoagulation | |
| 0.320 | 9 (17%) | 51 (23%) | 60 (22%) | ACE inhibitor | |
| 0.594 | 14 (26%) | 66 (30%) | 80 (29%) | ARB | |
| 0.862 | 6 (11%) | 23 (10%) | 29 (11%) | Calcium channel blocker | |
| 0.976 | 34 (64%) | 140 (64%) | 174 (64%) | ß-Blocking agent | |
| 0.987 | 36 (68%) | 149 (68%) | 185 (68%) | Loop diuretics | |
| 0.506 | 3 (6%) | 8 (4%) | 11 (4%) | Thiazide diuretics | |
| 0.452 | 27 (51%) | 99 (45%) | 126 (46%) | Potassium sparing diuretics | |
| 0.513 | 30 (57%) | 113 (52%) | 143 (53%) | Statins | |
| 0.322 | 0 (0%) | 4 (2%) | 4 (1%) | Fibrates | |
| 0.625 | 12 (23%) | 43 (20%) | 55 (20%) | Oral antidiabetic drugs | |
| 0.462 | 8 (15%) | 25 (11%) | 33 (12%) | Insulin | |
| 0.086 | 15 (28%) | 39 (18%) | 54 (20%) | Digitalis | |
| 0.812 | 22 (41%) | 87 (40%) | 109 (40%) | Nitrates | |
| **0.027** | 4 (7%) | 4 (2%) | 8 (3%) | Allopurinol | |
|  |  |  |  | **Laboratory parameters** | |
| 0.953 | 0.1 (0.1-0.1) | 0.1 (0.1-0.1) | 0.1 (0.1-0.1) | Troponin I (Mic gr/L) | |
| **0.020** | 7.5 (6.4-10.1) | 7.4 (6.1-8.9) | 7.4 (6.2-8.9) | WBC count (10^3/µL) | |
| **0.047** | 3.9 (3.5-4.8) | 4.5 (3.9-4.8) | 4.3 (3.8-4.8) | RBC count (10^6/ µL) | |
| **0.018** | 11.2 (9.5-12.5) | 11.9 (10.7-13.5) | 11.8 (10.5-13.5) | Hemoglobin (g/dL) | |
| **0.004** | 34 (29-37) | 37 (33-40) | 36 (32-40) | Hematocrit (%) | |
| 0.259 | 83 (77-88) | 85 (79-89) | 84 (78-89) | MCV (f lit) | |
| **0.027** | 27 (24-28) | 28 (25-30) | 28 (25-27) | MCH (Pg) | |
| **0.003** | 32 (31-33) | 33 (32-34) | 33 (32-34) | MCHC (g/dl) | |
| **p<0.001** | 16 (14-18) | 15 (14-16) | 15 (14-17) | RDW-CV (fl) | |
| 0.293 | 198 (151-235) | 186 (155-234) | 186 (155-234) | Platelet count (10^3/µL) | |
| **0.047** | 15 (14-18) | 14 (13-16) | 14 (13-16) | PT(sec) | |
| 0.200 | 34 (30-40) | 33 (30-38) | 33 (30-38) | PTT (sec) | |
| 0.171 | 1.4 (1.1-1.8) | 1.2 (1-1.5) | 1.2 (1.1-1.6) | INR (Index) | |
| 0.951 | 115 (101-149) | 122 (95-162) | 120 (96-160) | Random BS (mg/dL) | |
| **0.001** | 136 (133-140) | 139 (137-141) | 139 (136-141) | Sodium (mEq/dL) | |
| **0.001** | 4.5 (4.2-5) | 4.3 (4-4.6) | 4.3 (4-4.7) | Potassium (mEq/dL) | |
| **0.023** | 25 (19-40) | 21 (17-28) | 22 (18-30) | BUN (mg/dL) | |
| 0.395 | 1.3 (1-1.7) | 1.1 (0.9-1.4) | 1.2 (0.9-1.4) | Creatinine (mg/dL) | |
| **0.044** | 36 (21-54) | 22 (17-32) | 24 (18-38) [90] | SGOT (mg/dL) *** | |
| **0.018** | 39 (23-95) | 25 (13-37) | 26 (16-39) [90] | SGPT (IU/L) *** | |
| 0.619 | 186 (173-260) | 205 (155-262) | 199 (161-263) [86] | ALP (mg/dL) *** | |
| **0.006** | 3.5 (3.4-3.7) | 3.9 (3.6-4.2) | 3.8 (3.5-4.1) [76] | Albumin (mg/dL) *** | |
| **0.036** | 2.9 (2.3-3.3) | 2.5 (2.1-2.9) | 2.6 (2.1-2.9) [57] | Globulin (g/dL) *** | |
| 0.871 | 6.2 (5.8-6.9) | 6.5 (5.9-6.9) | 6.5 (5.8-6.9) [57] | Total protein (g/dL) *** | |
| 0.121 | 1.2 (1-3.4) | 1.1 (0.5-1.6) | 1.2 (0.5-1.7) [64] | Total bilirubin (mg/dL) *** | |
| 0.072 | 0.5 (0.4-2.1) | 0.3 (0.2-0.5) | 0.3 (0.2-0.6) [66] | Direct bilirubin (mg/dL) *** | |
| 0.074 | 71 (59-99) | 102 (76-127) | 96 (72-123) [101] | Triglyceride (mg/dL) *** | |
| **0.001** | 93 (81-107) | 138 (117-158) | 131 (102-156) [100] | Cholesterol (mg/dL) *** | |
| 0.572 | 28 (21-36) | 35 (29-43) | 35 (28-43) [94] | HDL-CH (mg/dL) *** | |
| **0.008** | 51 (44-53) | 74 (49-98) | 71 (48-93) [94] | LDL-C (mg/dL) *** | |
| **P<0.001** | 11.4 (9.8-12.7) | 7.7 (5.9-8.9) | 8.5 (6.8-9.9) [67] | Uric acid (mg/dL) *** | |
| 0.069 | 18 (11-30) | 15 (10-17) | 15 (11-20) [87] | Ck-mb (IU/L) ******* | |
| For abbreviations, please see Tables 1 and 2.  ^a^ Binary variables are expressed by number (percentage); continuous variables are illustrated as Median (first quartile-third quartile)  ^b^ Variables were compared using the Chi-square test, Student t test, and the Mann-Whitney U test for categorical variables, continuous variables with normal distribution, and non-normal distributions, respectively.  ^c^ All statistically significant p values (p < 0.05) are in bold.  * They were measured in patients with diabetes mellitus.  ** They were measured in patients with hypertension.  *** Shows laboratory parameters that were not requested for all patients. The number of patients from which statistics was calculated is shown in a bracket in front of the quartiles. | | | | | |
