# Supplementary figures and images for: Comparison of the red blood cell indices based on accuracy, sensitivity, and specificity to predict one-year mortality in heart failure patients
Source: BMC Cardiovasc Disord. 2022 Dec 7;22:532. doi: 10.1186/s12872-022-02987-x (PMC9727904; doi:10.1186/s12872-022-02987-x)

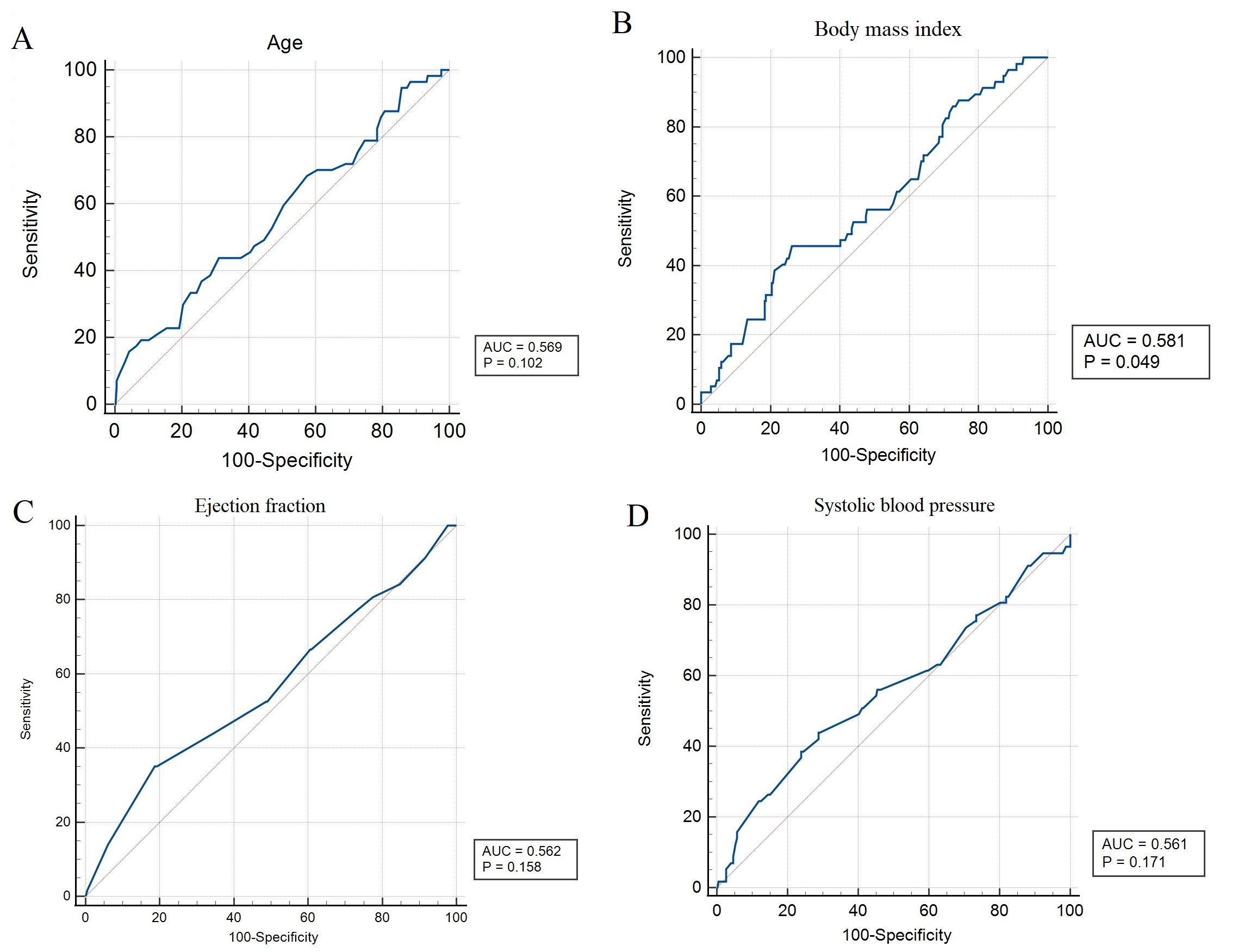

Supplement: Supplementary file 3 — Additional file 3: Fig. S1. The receiver operating characteristic (ROC) curves analyses were applied to predict mortality within the one-year follow-up for (A) Age, (B) Body mass index, (C) Ejection fraction, and (D) Systolic blood pressure [file 12872_2022_2987_MOESM3_ESM.jpg]
